# Differences in Factors Influencing Deprescribing between Primary Care Providers: Cross-Sectional Study

**DOI:** 10.3390/ijerph20064957

**Published:** 2023-03-11

**Authors:** Iva Bužančić, Maja Ortner Hadžiabdić

**Affiliations:** 1 Faculty of Pharmacy and Biochemistry, University of Zagreb, A. Kovačića 1, 10 000 Zagreb, Croatia; 2 City Pharmacies Zagreb, Kralja Držislava 6, 10 000 Zagreb, Croatia

**Keywords:** deprescribing, barrier, facilitator, primary healthcare, questionnaire

## Abstract

Deprescribing is a notable approach to improve medication management, but few healthcare systems recognize it. To introduce a new practice, it is important to examine the factors influencing the provision of a new or elaborate cognitive service within the desired setting. This study explores the perceived barriers and facilitators of deprescribing by primary healthcare providers, and identifies the factors associated with a willingness to suggest deprescribing. A cross-sectional survey was conducted (in Croatia, between October 2021 and January 2022) using a validated comprehensive healthcare providers’ opinions, preferences, and attitudes towards deprescribing (CHOPPED) questionnaire. A total of 419 pharmacists and 124 physicians participated. Participants showed a high willingness to deprescribe, with significantly higher scores in physicians than in pharmacists (5.00 (interquartile range—IQR 5–5) vs. 4.00 (IQR 4–5), *p* < 0.001). Pharmacists had significantly higher scores in seven out of ten factors (knowledge, awareness, collaboration facilitators, competencies facilitators, healthcare system facilitators, collaboration barriers, competencies barriers) while in the remaining three factors (patient facilitators, patient and healthcare system barriers) there was no difference in scores. The strongest positive correlation with willingness to suggest deprescribing was found with the collaboration and healthcare system facilitators factors for pharmacists (G = 0.331, *p* < 0.001, and G = 0.309, *p* < 0.001, respectively), and with knowledge, awareness, and patient facilitators factors for physicians (G = 0.446, *p* = 0.001; G = 0.771, *p* < 0.001; and G = 0.259, *p* = 0.043, respectively). Primary healthcare providers are willing to suggest deprescribing but face different barriers and facilitators. For pharmacists, the most important facilitators were extrinsic, while for physicians they were more intrinsic and patient related. The stated results provide target areas which one could focus upon to help to engage healthcare providers in deprescribing.

## 1. Introduction

Inappropriate polypharmacy (the use of five or more medicines concurrently), is a well-known risk factor for negative health outcomes, including increased healthcare costs, adverse drug events, drug interactions, decline in functional status, medication non-adherence, or cognitive impairment [1]. This problem is especially worrisome in regard to the elderly. As the global population is aging, it is expected that increasing rates of unnecessary polypharmacy will lead to inadequate care for a large number of sensitive elderly patients. Many countries are recognizing this problem and reforming policy paths in order to keep a sustainable healthcare system [2,3,4].

In recent years, deprescribing, the planned and supervised process of medication withdrawal or dose reduction with the intent to manage polypharmacy and improve outcomes [5], is becoming a noticeable approach to help healthcare providers resolve both existing and potential medication-related problems [6].

Different healthcare providers can uniquely contribute to deprescribing [7]. It has been shown that pharmacist-led or -initiated deprescribing interventions are successful and useful [8,9,10,11]. Pharmacists can identify candidates, initiate conversations on deprescribing, or suggest deprescribing interventions to physicians, as well as monitor and follow up on patients. Physicians’ knowledge and relationship with patients can contribute to the easier adoption of suggested deprescribing [12]. Even though deprescribing is considered a part of good prescribing practice, and good clinical or pharmaceutical care, in many healthcare settings and systems it is still a novel approach. For instance, at the time of the study there were no official workflows, guidelines, or recommendations regarding providing deprescribing at the primary healthcare level in Croatia, nor was deprescribing defined as a part of any diagnostic–therapeutic approach. There were no official data on the topic of deprescribing at any healthcare level, and healthcare providers were not reimbursed for deprescribing. Suggesting deprescribing and providing adequate follow-up to patients were still matters of healthcare professionals’ discretion. Both pharmacists and physicians suggest deprescribing to their patients as a part of their routine work, but this often depends on a collaborative approach. Due to a lack of access to the entirety of patients’ medical records and the inability to make significant inputs into electronic records, pharmacists contact the primary care physician to discuss comprehensive interventions. Primary care physicians often discuss deprescribing with fellow specialist doctors when it comes to specialist-prescribed medications. Similarly to Croatia, in the majority of European countries, proactive deprescribing policies, frameworks, and workflows are still being formed [13]. Evidence is being gathered on how to successfully identify the challenges of implementation of deprescribing across healthcare systems and settings [14,15], as well as how to enhance deprescribing interventions both in research and in everyday practice [16,17].

In order to introduce a new practice, it is important to examine potential barriers and facilitators within the desired setting. Exploring opinions and attitudes towards new or elaborate cognitive services often requires time, experienced researchers, financial support, and readily available participants. To help reduce the costs of research and reach a larger number of important stakeholders who will provide the service and whose opinions, perceptions, experiences, and attitudes effect the provision of a service, a validated tool can be used for the identification of barriers and facilitators for deprescribing.

The aim of this study was to explore the perceived barriers and facilitators of deprescribing by primary care physicians and pharmacists inexperienced in every day deprescribing, and to identify whether any factors were associated with a willingness to suggest deprescribing.

## 2. Materials and Methods

A cross-sectional online survey was used to collect data, with LimeSurvey^®^ software being used in the design and the distribution of the survey (LimeSurvey Version 2.67.1 + 170626; LimeSurvey GmbH, Hamburg, Germany. URL: http://www.limesurvey.org, accessed on 15 September 2021). The software used is part of the services, data, and collaboration system tools available from the University of Zagreb Computing Centre (SRCE). The survey consisted of three parts: sociodemographic questions, a comprehensive healthcare provider’s opinions, preferences, and attitudes towards deprescribing questionnaire (CHOPPED), and a case vignette (analysis not included herein). The development and validation of the CHOPPED questionnaire have been described elsewhere [18]. Two versions of the questionnaire are available, one for pharmacists (with 38 items) and one for physicians (with 36 items). Each questionnaire consists of ten factors, knowledge, awareness, patient barriers/facilitators, competencies barriers/facilitators, collaboration barriers/facilitators, and healthcare system barriers/facilitators, and one question regarding the willingness to suggest deprescribing. Items within the knowledge factor, awareness factor, and willingness to suggest deprescribing are equal in both versions. For each facilitators factor, there was a difference in one item between the versions. For the barriers factor, the difference between the two versions was in one item for all factors except for the collaboration barrier factor, which had three profession-specific items that were unique in each version. All of the questions within the CHOPPED questionnaire were scored on a 5-point Likert scale (“strongly disagree”, “disagree”, “neither agree nor disagree”, “agree”, and “strongly agree”). Details on items and the differences between the two versions are available in Supplementary File S1.

The link to the survey was sent to community pharmacists and primary care physicians (general practitioners or family physicians) as their professional email addresses were publicly available on the national chambers of pharmacists and physicians. Participants were asked to forward the link to the survey to potential participants (*snowballing method*). At the beginning of the survey, the participants had to read and digitally authorize the informed consent, without which they could not access the survey. All data were collected anonymously. Participants could save the answers of the unfinished survey and complete it at a later time. Two reminders to complete the survey were sent four and eight weeks after the initial email. The study was conducted in Croatia, between October 2021 and January 2022. To ensure there were no duplicate inputs, each unique IP address was marked in the responses. If a single IP address had multiple inputs, they were cross-checked for the uniqueness of the socio-demographic information. Duplicate unfinished or answerless questionnaire entries from duplicate IP addresses were discarded, as were other invalid or incomplete inputs. 

Since there were no data or studies in Croatia on the topic of healthcare providers’ attitudes towards deprescribing, a single population proportion formula was used, with a 95% confidence level and relative precision of 5%, and the proportion of primary care providers willing to suggest deprescribing was 50%. Sample size was determined based on the number of registered primary care physicians and community pharmacists. Data were available from the Croatian health statistics yearbook and the Croatian chamber of pharmacists states than in 2021 there were 2180 registered primary care physicians and 2870 registered community pharmacists [19,20]. Therefore, the calculated sample size was 339 for pharmacists and 327 for physicians.

Sociodemographic data were analyzed using descriptive statistics. The factor score was calculated by summing the score of each item and dividing it by the number of items within the factor. A chi-squared test was used to analyze differences in frequencies between groups. The Mann–Whitney U test was used to determine differences in CHOPPED factor scores between professions. Gamma rank correlation was used to analyze potential associations between factor scores and the willingness to deprescribe (using ordinal data for both factors’ scores and willingness to suggest deprescribing). For all analyses, a value of *p* < 0.05 was considered to be statistically significant. All analyses were performed using IBM SPSS Statistics for Windows, Version 26.0. (IBM Corp., Armonk, NY, USA). 

## 3. Results

In total, 419 pharmacists’ and 124 physicians’ inputs were available for analysis. No statistically significant differences, in any characteristics, were found for both the pharmacists’ and physicians’ samples when comparing those who completed the survey and those who completed just a part of it.

### 3.1. Participants Characteristics

Healthcare providers who participated in the study were mostly female at 82.32% (86.62% among pharmacists and 74.30% among physicians). They had a median of 36 years of age (interquartile range (IQR) 29–48), and a median of 11 years of professional experience (IQR 4–22). More than half of them worked in an urban area (55.99%), 32.04% worked in suburban areas, and 11.97% provided healthcare services in rural areas across Croatia. Detailed characteristics of participants and differences between professions can be found in Table 1. Practices were almost equally placed either near other healthcare facilities (34.26%) or defined as a displaced/standalone practice (35.54%), while 25.78% of practices were within another larger healthcare facility, and 4.42% were within a shopping center.

### 3.2. Knowledge and Awareness of Deprescribing

When it came to knowledge about deprescribing, pharmacists were more likely to agree with statements that deprescribing involves tapering and reducing the dose of a medication or that it represents changing medication to a safer alternative than physicians (71.59% vs. 52.42%, χ^2^(4) = 25.64, *p* < 0.001, and 61.09% vs. 43.49%, χ^2^(4) = 15.16, *p* = 0.004, respectively). 

Even though the majority of all healthcare providers agreed with all of the statements in the awareness factor, pharmacists were more likely to find deprescribing as being as important as prescribing medication compared to physicians (94.98% vs. 83.87% χ^2^(4) = 17.64, *p* = 0.001). Physicians were less likely to be aware of the deprescribing benefits, including a reduction in healthcare expenditures, an improvement in outcomes, or adherence, than pharmacists (91.88% vs. 75.86%, χ^2^(4) = 40.02, *p* < 0.001, 86.87% vs. 66.13%, χ^2^(4) = 35.05, *p* < 0.001, and 80.44% vs. 77.41% χ^2^(4) = 29.01, *p* < 0.001, respectively).

### 3.3. Facilitators and Barriers of Deprescribing

Four factors pertaining to the facilitators and barriers of deprescribing were examined: patient, collaboration, competencies, and healthcare system. 

In the patient facilitators factor, participants agreed the most with the statement “*I am keener to suggest stopping medications to patients who show greater involvement in their medication*” with 83.74% being positive answers. Conversely, only 39.22% of participants agreed that they would suggest deprescribing to patients if patients expressed their desire to have their number of medications reduced. There was no difference in agreement between professions for any of the statements.

In the collaboration facilitators factor, both professions agreed that a public healthcare project on deprescribing would be encouraging (81.30% of all participants). In the physicians’ version, 77.19% considered having pharmacists’ evidence-based deprescribing rationale to be useful for suggesting deprescribing, and more than half (57.89%) found a close collaboration with a pharmacist encouraging. A greater majority of pharmacists agreed that a close collaboration with a physician would encourage them to suggest deprescribing (92.59%).

The majority of participants (85.34 of physicians and 89.82% of pharmacists) agreed that they needed incentives when it came to their competencies. Physicians were less likely to state that they needed education on medication review, how to approach patients regarding deprescribing, or guidelines and algorithms than pharmacists (69.45% vs. 93.32%, χ^2^(4) = 62.69, *p* < 0.001; 70.83% vs. 87.83%, χ^2^(4) = 34.19, *p* < 0.001; 69.44% vs. 92.36%, χ^2^(4) = 52.39, *p* < 0.001, respectively). 

Even though more than 60% (64.15%) of all participants believed that reimbursement for deprescribing is needed, physicians were more likely to disagree with this statement than pharmacists (36.11% vs. 13.60%, χ^2^(4) = 42.53, *p* < 0.001). There was no difference in agreement between professions for other items in the healthcare system facilitators factor.

In the patient barriers factor, healthcare providers were the least worried about deprescribing suggestions negatively influencing their relationship with patients, with 15.04% of participants agreeing with this statement. On the other hand, the majority of healthcare providers (83.69%) found it difficult to suggest deprescribing to patients with low involvement in medication decision-making. Pharmacists were more likely to find this statement to be a barrier than physicians (88.07% vs. 49.06%, χ^2^(4) = 58.73, *p* < 0.001).

In the collaboration barriers factor, pharmacists were most concerned about physicians finding their suggestions inappropriate (70.80%), while physicians found the biggest barrier being a lack of real-time communication with other healthcare providers (70.64%).

Competencies barriers differed amongst healthcare providers. Pharmacists found recommending deprescribing preventative medications a higher barrier than physicians (44.63% vs. 13.21%, χ^2^(4) = 38.46, *p* < 0.0001). Pharmacists indicated that they had lower confidence and found it more difficult to identify potentially inappropriate medications than physicians (32.22% vs. 13.20%, χ^2^(4) = 12.46, *p* = 0.014, and 57.28% vs. 33.96%, χ^2^(4) = 11.94, *p* = 0.018, respectively).

Within a healthcare system, participants perceived a lack of time and lack of legislation as being the biggest barriers (72.25% and 78.39%, respectively). Physicians were less hindered by a lack of legislation than pharmacists (49.06% vs. 82.10%, χ^2^(4) = 43.52, *p* < 0.001). Pharmacists considered a lack of time to be a higher obstacle than physicians (74.46% vs. 54.72%, ^χ2^(4) = 10.80, *p* = 0.029).

Detailed answers to all of the questions within both versions of the CHOPPED questionnaire can be seen in Supplementary Figure S1.

**Figure 1:**
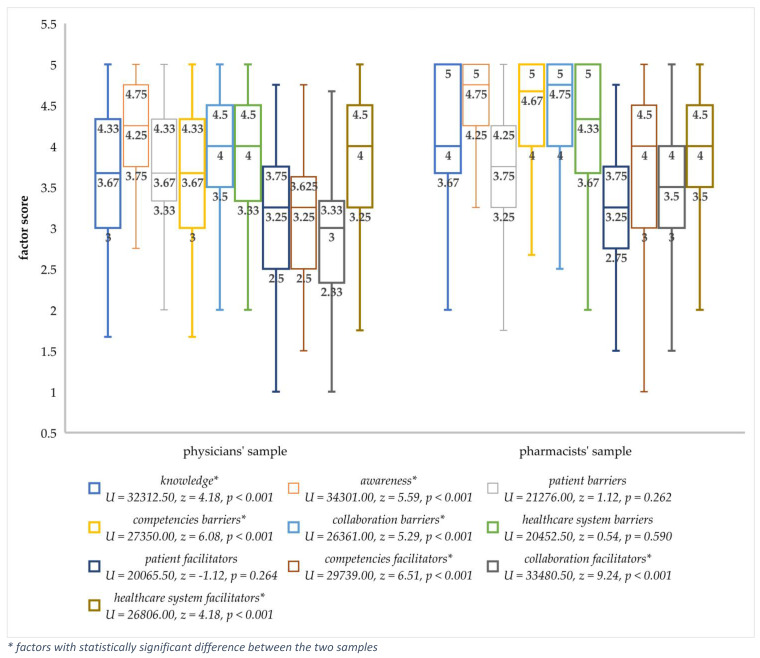
Differences in factor scores between pharmacists and physicians (Mann–Whitney U test).

When analyzing differences in factor scores between pharmacists and physicians, it was found that pharmacists had statistically significantly higher scores in all factors except for patient facilitators, patient barriers, and healthcare system barriers (Figure 1).

### 3.4. Willingness to Suggest Deprescribing

More than 80% (*n* = 473, 87.12%) of all healthcare providers stated that they would suggest deprescribing to a patient if this was appropriate. Pharmacists were more likely to show uncertainty, with 11.97% of them stating “neither agree nor disagree” in comparison to 3.36% of physicians (χ^2^(4) = 44.93, *p* < 0.001). In both the pharmacists’ and the physicians’ samples, there was no difference in willingness to suggest deprescribing between participants based on age, years of experience, education, location, or practice characteristics. The median willingness to suggest deprescribing score was statistically significantly higher in physicians than in pharmacists (5.00 (IQR 5–5) vs. (4.00 (IQR 4–5)), U = 15293.00, z = −6.62, *p* < 0.001).

**Figure 2:**
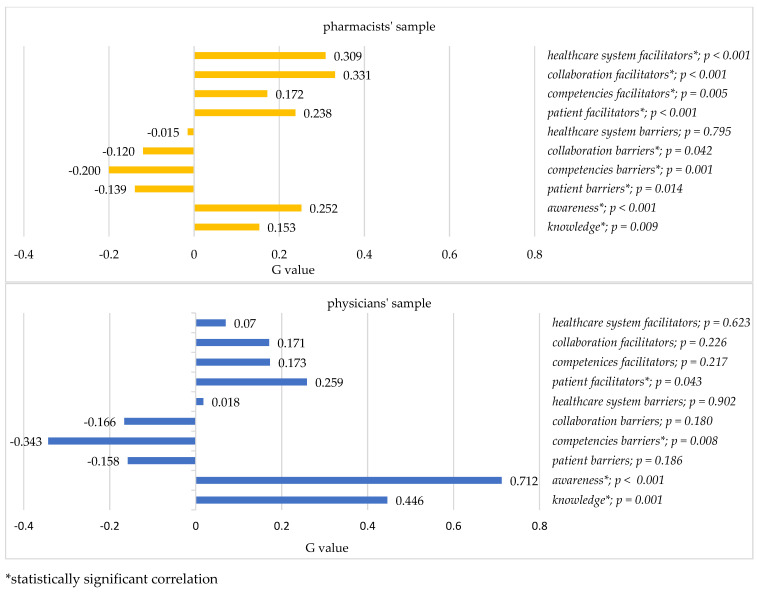
Gamma rank correlation.

Several factors were associated with an increased willingness to suggest deprescribing in both samples. In the pharmacists’ sample, all factors, except healthcare system barriers, were statistically significantly associated with a willingness to suggest deprescribing. The strength of association was weak to moderate, with collaboration facilitator and healthcare system facilitator factors showing the strongest correlation in the pharmacists’ sample. In the physicians’ sample, four factors were associated with a willingness to suggest deprescribing. The knowledge factor was very strongly associated with a willingness to suggest deprescribing, while the awareness factor exhibited a strong correlation. Patient facilitators and competencies barriers factors were moderately associated with a wiliness to suggest deprescribing. The negative correlation found between a willingness to suggest deprescribing and the competencies barriers factor indicates that an increased perception of a lack of personal competencies was associated with a lower willingness to suggest deprescribing. Additional information on the correlation between the willingness to suggest deprescribing and the CHOPPED factors can be found in Figure 2.

## 4. Discussion

Healthcare providers are willing to suggest deprescribing, with pharmacists showing more uncertainty than physicians. Contrariwise, pharmacists showed higher knowledge and awareness of the deprescribing benefits than physicians. An Irish study on community pharmacists highlights a similar finding, where pharmacists express high knowledge, but their willingness is hindered by different obstacles [21]. Pharmacists’ uncertainty could be attributed to the lower confidence that they expressed in comparison to physicians. These results are in line with research confirming that the majority of physicians feel comfortable and self-assured with deprescribing [22,23], as well as that pharmacists often feel less confident in their role in deprescribing [24].

There is a noticeable distinction between pharmacists and physicians when it comes to the correlation between the willingness to suggest deprescribing and other factors. For pharmacists, it was the collaboration facilitators and healthcare system facilitators factors, and for physicians it was knowledge, awareness, and patient facilitators factors. This not only accentuates the differences between professions, but also highlights the possible target areas which one could focus upon to help to engage healthcare providers in deprescribing. 

Each healthcare system is different and needs a customized implementational strategy in order to deliver an intervention. Analyzing determinants which affect this implementation is a critical step in ensuring the success of a clinical intervention [25]. The decision to suggest deprescribing is complex and influenced by a number of factors, and some of which can be overlooked when only explored through the viewpoint of those involved in qualitative studies. Having a tool which can help measure the willingness to deprescribe in a larger number of healthcare providers can give a more realistic insight into the readiness of the setting to implement deprescribing. Tools similar to the CHOPPED questionnaire are being developed, which underlines the need for and importance of exploring deprescribing factors within healthcare systems [26,27]. Most qualitative research has focused on the opinions, beliefs, and attitudes of physicians, with pharmacists and other healthcare providers being less represented [22,28,29,30,31,32,33,34,35]. It is sensible to involve those who prescribe medication in deprescribing; nevertheless, research shows that other healthcare providers can be important stakeholders and facilitators of deprescribing [10,11,36]. Deprescribing is an intricate intervention often more successful if a multidisciplinary approach is satisfied [37,38]. The CHOPPED questionnaire explores, among others, collaboration barriers and facilitators in both versions, with the increased collaboration facilitators factor score being associated with an increased willingness to suggest deprescribing in the pharmacists’ version for this sample. Furthermore, increased knowledge and awareness of deprescribing were associated with an increased willingness to suggest deprescribing, while a decreased perception of competencies was associated with a decreased willingness to deprescribe in both versions. Continuous professional education and early introduction to the concept of deprescribing as a part of pharmacy or medical curricula could help future generations of pharmacists and physicians to increase their involvement in proactive deprescribing [8,39,40]. Taking actions to involve patients or the public in deprescribing, such as having open discussion days on medication optimization actions with patient advocacy groups or visiting nursing homes to talk to patients about healthcare, can have multiple benefits. On the one hand it can increase the patient facilitator factor score which is important for healthcare providers to increase their willingness to suggest deprescribing, and on the other hand it creates opportunities for patients to help guide healthcare providers in creating interventions tailored to their specific needs. Finally, the CHOPPED questionnaire as a tool has the potential to help characterize differences in factors influencing deprescribing among healthcare providers and to help recognize target areas needing improvement.

### 4.1. Limitations

Several limitations of this study should be stated. Nonresponse bias, as a type of selection bias when using an online survey as the method of data collection, could be viewed as a limitation of this study. The true response rate could not be determined since the exact number of healthcare providers reached by the snowballing method is unknown. Additionally, unknown number of email addresses could have been incorrect, duplicated, or unavailable. Differences in sample sizes, age and experience of participants, or female-dominated participation could be viewed as a shortcoming. Regardless, selection bias can be considered to be minimal since the characteristics of both samples of involved healthcare providers (gender, educational attainment, and practice location) correspond to those of the population of interest [19]. The survey reached around 15% of the overall community of the pharmacists’ population and around 6% of the population of primary care physicians in Croatia, which can be considered satisfactory, especially in the circumstances of the pandemic. Based on the number of respondents, the crude estimation of the targeted population for each profession, and the defined confidence interval of 95%, a satisfactory margin of error was determined: 4.43% for the pharmacists’ sample and 8.56% for the physicians’ sample. The results of this study could not be generalized and do not apply to different study populations aside from ours. The study was carried out in Croatia, a small country with a developing healthcare system based on social solidarity. It is important to state that deprescribing is very new concept in the investigated setting and this may have led to healthcare providers being more familiar with the concept being the ones willing to participate in the study, creating a bias. Nevertheless, a significant advantage of this research is a strong methodology for the development and validation of the tool used in this research with confirmed face, content, construct, and criterion validity [18]. 

### 4.2. Implications for Research Practice

One advantage of CHOPPED as a tool is that it can be reused to re-evaluate healthcare professionals’ viewpoints as providers as the system changes or adapts. The two versions of the tool allow for a more tailored approach to each profession, yet the universal and shared factors ensure that systematic changes can be achieved where necessary. Optimizing workload, giving access to important patient information, providing patient deprescribing materials, improving knowledge on the advantages of deprescribing, and creating opportunities for inter- and intra-professional communication and collaboration are the possible goals for overcoming the hurdles recognized by the CHOPPED tool. For instance, within a health center, a multidisciplinary workshop focusing on increasing collaborative practice and awareness on deprescribing benefits could be of help to both professions. Studies including more mature pharmacists, a larger sample of physicians, and healthcare providers less interested or aware of deprescribing, as well as studies in countries with different types of healthcare systems and different experiences with deprescribing interventions, should be carried out to additionally confirm the appropriateness and usefulness of the CHOPPED questionnaire. Future research can combine the use of the CHOPPED questionnaire with a model intervention to investigate whether or not the tool can help to identify obstacles prior to and during deprescribing.

## 5. Conclusions

Primary care physicians and pharmacists are willing to suggest deprescribing but are faced with different barriers and facilitators. For pharmacists, the most important facilitators were extrinsic factors (collaboration and healthcare-system-related), while for physicians these were more intrinsic (knowledge and awareness) and patient-related. This study highlights the differences in determinants influencing deprescribing between professions, and also accentuates the possible target areas upon which one could focus to help to engage healthcare providers in deprescribing.

## Figures and Tables

**Table 1:** Participants’ characteristics.

Characteristic	Pharmacists (*n* = 419)	Physicians (*n* = 124)
sex (female gender, %)	86.62	74.30
age (median, IQR)	35 (28–43)	50 (33–60)
years of experience (median, IQR)	10 (3–19)	24 (6.75–33)
highest educational attainment		
graduate degree	83.94	26.16
postgraduate specialist course ^a^	11.44	n/a
health specialization ^b^	3.41	72.80
master’s degree (MSc)	0.48	0.72
doctoral degree (PhD)	0.73	0.32
location		
urban	59.85	43.58
suburban	31.15	38.54
rural	9.00	17.88
practice location		
within another healthcare facility	16.55	59.78
near another healthcare facility	40.15	11.17
within a shopping center	5.59	n/a
displaced/not near another healthcare facility	37.71	29.05
practice ownership		
private/concession	54.00	23.20
public	46.00	76.80
type of ownership		
single practice	13.12	n/a
chain pharmacy <10 units	28.00	n/a
chain pharmacy >10 units	58.88	n/a
number of patients in practice (median, IQR)	n/a	1650 (1250–1946)
percentage of elderly patients in practice (median, IQR)	n/a	35 (28.75–50)

^a^ 1-year course, ^b^ 3-year healthcare residency including postgraduate specialist course, IQR—interquartile range; n/a- not applicable.

## Data Availability

The datasets used and/or analyzed during the current study are available from the corresponding author upon reasonable request.

## Supplementary Materials

The following supporting information can be downloaded at https://www.mdpi.com/article/10.3390/ijerph20064957/s1: File S1: Questions within the CHOPPED questionnaire; Figure S1: Answers to the CHOPPED questionnaire. All authors have read and agreed to the published version of the manuscript.
