# Supplementary material for: Differences in Factors Influencing Deprescribing between Primary Care Providers: Cross-Sectional Study
Source: Int J Environ Res Public Health. 2023 Mar 11;20(6):4957. doi: 10.3390/ijerph20064957 (PMC10049550; doi:10.3390/ijerph20064957)
Supplement: Supplementary file 1 [file ijerph-20-04957-s001.zip › Supplementary Figure S1.pdf]

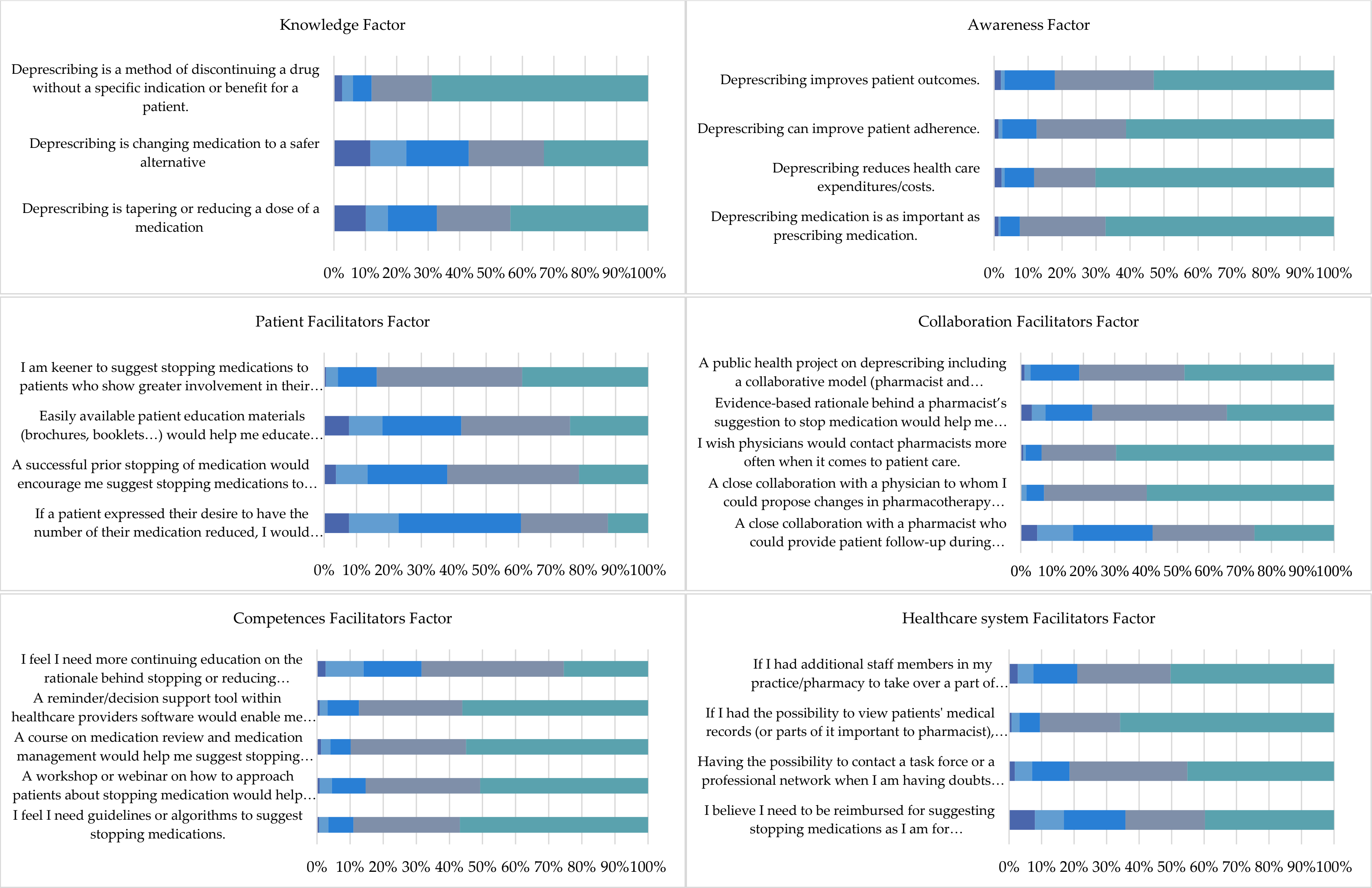

Knowledge Factor
Deprescribing is a method of discontinuing a drug without a specific indication or benefit for a patient.
Deprescribing is changing medication to a safer alternative
Deprescribing is tapering or reducing a dose of a medication
0% 10% 20% 30% 40% 50% 60% 70% 80% 90% 100%
Awareness Factor
Deprescribing improves patient outcomes.
Deprescribing can improve patient adherence.
Deprescribing reduces health care expenditures/costs.
Deprescribing medication is as important as prescribing medication.
0% 10% 20% 30% 40% 50% 60% 70% 80% 90% 100%
Patient Facilitators Factor
I am keener to suggest stopping medications to patients who show greater involvement in their…
Easily available patient education materials (brochures, booklets…) would help me educate…
A successful prior stopping of medication would encourage me suggest stopping medications to…
If a patient expressed their desire to have the number of their medication reduced, I would…
0% 10% 20% 30% 40% 50% 60% 70% 80% 90% 100%
Collaboration Facilitators Factor
A public health project on deprescribing including a collaborative model (pharmacist and…
Evidence-based rationale behind a pharmacist's suggestion to stop medication would help me…
I wish physicians would contact pharmacists more often when it comes to patient care.
A close collaboration with a physician to whom I could propose changes in pharmacotherapy…
A close collaboration with a pharmacist who could provide patient follow-up during…
0% 10% 20% 30% 40% 50% 60% 70% 80% 90% 100%
Competences Facilitators Factor
I feel I need more continuing education on the rationale behind stopping or reducing…
A reminder/decision support tool within healthcare providers software would enable me…
A course on medication review and medication management would help me suggest stopping…
A workshop or webinar on how to approach patients about stopping medication would help…
I feel I need guidelines or algorithms to suggest stopping medications.
0% 10% 20% 30% 40% 50% 60% 70% 80% 90% 100%
Healthcare system Facilitators Factor
If I had additional staff members in my practice/pharmacy to take over a part of…
If I had the possibility to view patients' medical records (or parts of it important to pharmacist),…
Having the possibility to contact a task force or a professional network when I am having doubts…
I believe I need to be reimbursed for suggesting stopping medications as I am for…
0% 10% 20% 30% 40% 50% 60% 70% 80% 90% 100%

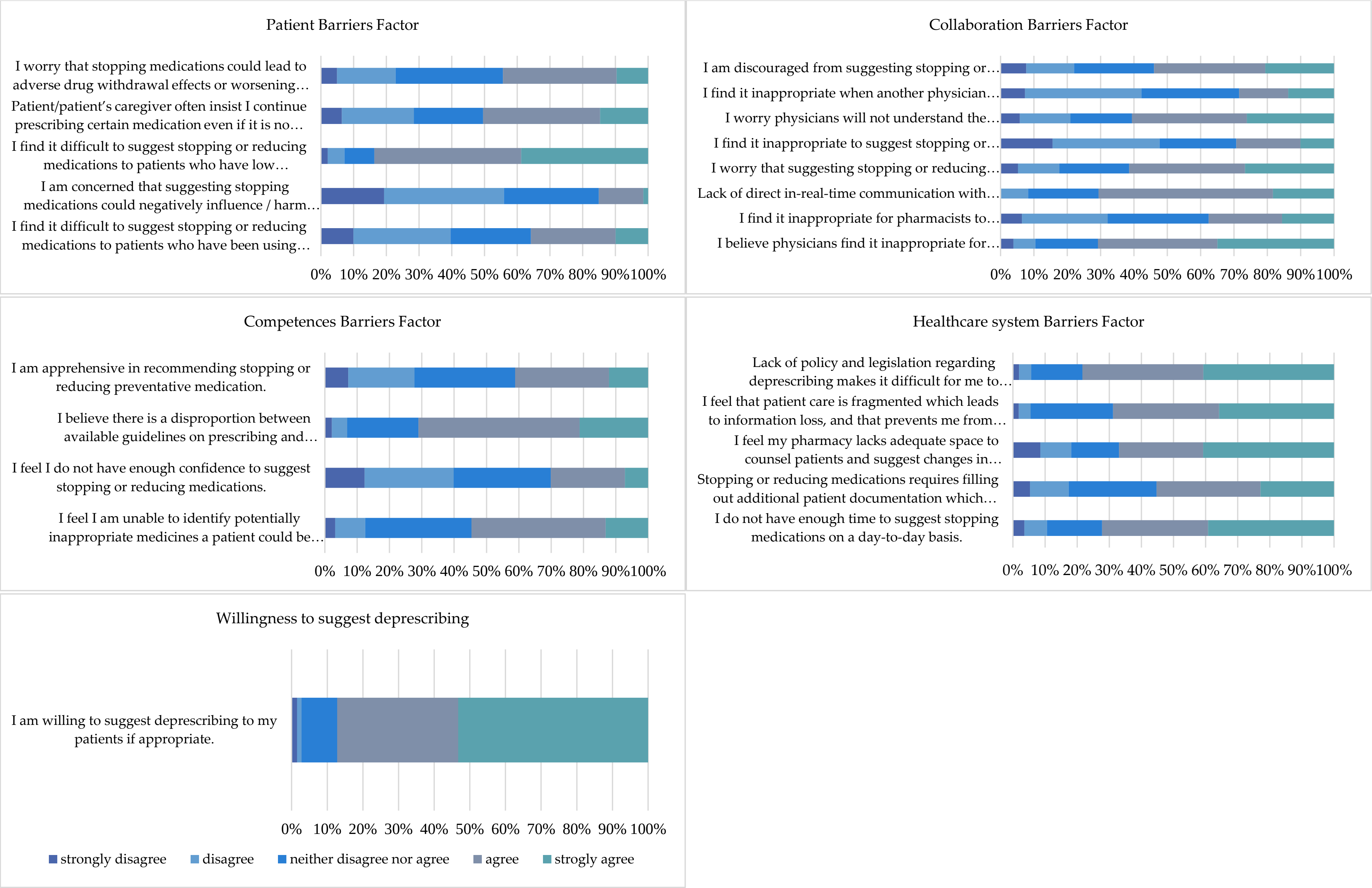

Supplementary Figure S1 Answers to the CHOPPED questionnaire
