# Supplementary material for: Differences in Factors Influencing Deprescribing between Primary Care Providers: Cross-Sectional Study
Source: Int J Environ Res Public Health. 2023 Mar 11;20(6):4957. doi: 10.3390/ijerph20064957 (PMC10049550; doi:10.3390/ijerph20064957)
Supplement: Supplementary file 1 [file ijerph-20-04957-s001.zip › Supplementary File S1.pdf]

Supplementary File S1 Questions within the CHOPPED questionnaire

SOCIODEMOGRAPHIC QUESTIONS

1. Sex:
   a) M
   b) F
   c) Do not wish to answer

2. Age (in years):
   a) __________

3. Work experience (in years)
   a) __________

4. Pharmacy where you practice is situated in/ Your practice is situated in:
   a) an urban area (metropolitan area, within a big city)
   b) suburban area (small town, outside of a bigger city)
   c) a rural area

5. Educational attainment:
   a) Graduate degree
   b) Postgraduate specialist degree in clinical pharmacy
   c) Other postgraduate specialist degree
   d) health specialisation (i.e. clinical pharmacy specialist)
   e) Masters' degree (Master of Science)
   f) PhD

6. Pharmacy where you work is:
   a) independent/ single owner pharmacy (one owner)
   b) within a small pharmacy chain (up to 10 pharmacies)
   c) within a large retail pharmacy chain (10 or more pharmacies)
   d) _______________

7. Pharmacy where you work/ Your practice is:
   a) is privately owned (person or a company)/ concession
   b) is government owner.

8. Pharmacy where you work is:
   a) within a clinic or a physician's practice.
   b) near a hospital, healthcare centre, or physician's practice.
   c) within a shopping centre or store.
   d) not near any other healthcare facilities.

9. Your practice is (GP version):
   a) a part of a larger clinic or health centre
   b) in a standalone object, dislocated from other healthcare providers
   c) near a hospital, healthcare centre, or other physician's practice.

10. Approximate number of patients you treat (GP version):
   a) _________________

11. What percentage of your patients are elderly individuals (older than 65 years) (GP version)
   a) __________________%

PHARMACISTS' VERSION:

1	Deprescribing is tapering or reducing a dose of a medication/ is a method of reducing drug dosage.
2	Deprescribing is changing medication to a safer alternative / a method of changing a drug to a safer alternative.
3	Deprescribing is a method of discontinuing a drug without a specific indication or benefit for a patient.
4	Deprescribing medication is as important as prescribing medication.
5	Deprescribing reduces health care expenditures/costs.
6	Deprescribing can improve patient adherence.
7	Deprescribing improves patient outcomes.
8	If a patient expressed their desire to have the number of their medication reduced, I would suggest stopping medications.
9	A successful prior stopping of medication would encourage me suggest stopping medications to a patient again.
**10***	Easily available patient education materials (brochures, booklets…) would help me educate and suggest stopping medications to my patients more.
11	I am keener to suggest stopping medications to patients who show greater involvement in their medication.
12	A close collaboration with a physician to whom I could propose changes in pharmacotherapy would encourage me to suggest stopping medications more.
**13***	I wish physicians would contact pharmacists more often when it comes to patient care.
14	A public health project on deprescribing including a collaborative model (pharmacist and physicians) would encourage me to suggest deprescribing more.
15	I feel I need guidelines or algorithms to suggest stopping medications.
16	A workshop or webinar on how to approach patients about stopping medication would help me suggest such changes more.
17	A course on medication review and medication management would help me suggest stopping or reducing medications more.
**18***	A reminder/decision support tool within healthcare providers software would enable me to suggest stopping medications more.
19	I believe I need to be reimbursed for suggesting stopping medications as I am for dispensing/other clinical services I provide.
**20***	If I had the possibility to view patients' medical records (or parts of it important to pharmacists), I would be able to suggest stopping or reducing medications more.
21	If I had additional staff members in my practice/pharmacy to take over a part of administrative workload, I would be able to suggest stopping or reducing medications more.
22	I find it difficult to suggest stopping or reducing medications to patients who have been using them for a long time even if they no longer needed them.
23	I am concerned that suggesting stopping medications could negatively influence / harm my relationship with my patient.
24	I find it difficult to suggest stopping or reducing medications to patients who have low understanding of their therapy or to those that have low involvement in medication decision-making.
**25***	I worry that stopping medications could lead to adverse drug withdrawal effects or worsening of patient's health.
26	I believe physicians find it inappropriate for pharmacists to suggest stopping or reducing medications.
**27***	I worry that suggesting stopping or reducing medications could negatively influence my relationship with prescribers.
**28***	I worry physicians will not understand the rationale behind my suggestions.
**29***	I am discouraged from suggesting stopping or reducing medications because I feel physicians would find my recommendations unknowledgeable.
30	I feel I am unable to identify potentially inappropriate medicines a patient could be taking that require stopping or reducing
31	I feel I do not have enough confidence to suggest stopping or reducing medications.
**32***	I believe there is a disproportion between available guidelines on prescribing and stopping medications which makes it difficult for me to suggest deprescribing.
33	I am apprehensive in recommending stopping or reducing preventative medication.
34	I do not have enough time to suggest stopping medications on a day-to-day basis.
35	Stopping or reducing medications requires filling out additional patient documentation which thwarts from suggesting such changes regularly.
**36***	I feel my pharmacy lacks adequate space to counsel patients and suggest changes in pharmacotherapy.
37	Lack of policy and legislation regarding deprescribing makes it difficult for me to suggest stopping or reducing medications.
38	I am willing to suggest deprescribing to my patients if appropriate.

PHYSICIANS' VERSION:

1	Deprescribing is tapering or reducing a dose of a medication/ is a method of reducing drug dosage.
2	Deprescribing is changing medication to a safer alternative / a method of changing a drug to a safer alternative.
3	Deprescribing is a method of discontinuing a drug without a specific indication or benefit for a patient.
4	Deprescribing medication is as important as prescribing medication.
5	Deprescribing reduces health care expenditures/costs.
6	Deprescribing can improve patient adherence.
7	Deprescribing improves patient outcomes.
8	If a patient expressed their desire to have the number of their medication reduced, I would suggest stopping medications.
9	A successful prior stopping of medication would encourage me suggest stopping medications to a patient again.
10	I am keener to suggest stopping medications to patients who show greater involvement in their medication.
11	A close collaboration with a pharmacist who could provide patient follow-up during medication withdrawal would encourage me to suggest stopping medications more.
12	Evidence-based rationale behind a pharmacist's suggestion to stop medication would help me accept pharmacist's suggestions.
13	A public health project on deprescribing including a collaborative model (pharmacist and physicians) would encourage me to suggest deprescribing more.
**14***	I feel I need more continuing education on the rationale behind stopping or reducing medication to be able to suggest such changes.
15	I feel I need guidelines or algorithms to suggest stopping medications.
16	A workshop or webinar on how to approach patients about stopping medication would help me suggest such changes more.
17	A course on medication review and medication management would help me suggest stopping or reducing medications more.
18	I believe I need to be reimbursed for suggesting stopping medications as I am for dispensing/other clinical services I provide.
**19***	Having the possibility to contact a task force or a professional network when I am having doubts regarding stopping or reducing medications, would encourage me to suggest such changes.
20	If I had additional staff members in my practice/pharmacy to take over a part of administrative workload, I would be able to suggest stopping or reducing medications more.
21	I find it difficult to suggest stopping or reducing medications to patients who have been using them for a long time even if they no longer needed them.
22	I am concerned that suggesting stopping medications could negatively influence / harm my relationship with my patient.
23	I find it difficult to suggest stopping or reducing medications to patients who have low understanding of their therapy or to those that have low involvement in medication decision-making.
**24***	Patient/patient's caregiver often insist I continue prescribing certain medication even if it is no longer necessary
25	I find it inappropriate for pharmacists to suggest stopping or reducing medications to my patients.
**26***	Lack of direct in-real-time communication with other healthcare providers (hospital doctors or specialists, pharmacist, nursing home care teams...) makes it difficult for me to suggest stopping or reducing medications.
**27***	I find it inappropriate to suggest stopping or reducing medications other physicians (other specialists) have prescribed.
**28***	I find it inappropriate when another physician suggests stopping medications I have prescribed.
29	I feel I am unable to identify potentially inappropriate medicines a patient could be taking that require stopping or reducing
30	I feel I do not have enough confidence to suggest stopping or reducing medications.
31	I am apprehensive in recommending stopping or reducing preventative medication.
32	I do not have enough time to suggest stopping medications on a day-to-day basis.
33	Stopping or reducing medications requires filling out additional patient documentation which thwarts from suggesting such changes regularly.
**34***	I feel that patient care is fragmented which leads to information loss, and that prevents me from suggesting major changes in patient's pharmacotherapy (such as stopping medications).
35	Lack of policy and legislation regarding deprescribing makes it difficult for me to suggest stopping or reducing medications.
36	I am willing to suggest deprescribing to my patients if appropriate.

***profession unique/specific items**
